# An Anti-Electromagnetic Attack PUF Based on a Configurable Ring Oscillator for Wireless Sensor Networks

**DOI:** 10.3390/s17092118

**Published:** 2017-09-15

**Authors:** Zhaojun Lu, Dongfang Li, Hailong Liu, Mingyang Gong, Zhenglin Liu

**Affiliations:** 1 School of Optical and Electronic Information, Huazhong University of Science and Technology, Wuhan 430074, China; D201377521@hust.edu.cn (Z.L.); D201577539@hust.edu.cn (H.L.); D201677550@hust.edu.cn (M.G.); 2 Beijing Institute of Computer Technology and Applications, Beijing 100854, China; lidongfang@hust.edu.cn

**Keywords:** wireless sensor network, configurable RO PUF, hardware efficiency, anti-EM attack

## Abstract

Wireless sensor networks (WSNs) are an emerging technology employed in some crucial applications. However, limited resources and physical exposure to attackers make security a challenging issue for a WSN. Ring oscillator-based physical unclonable function (RO PUF) is a potential option to protect the security of sensor nodes because it is able to generate random responses efficiently for a key extraction mechanism, which prevents the non-volatile memory from storing secret keys. In order to deploy RO PUF in a WSN, hardware efficiency, randomness, uniqueness, and reliability should be taken into account. Besides, the resistance to electromagnetic (EM) analysis attack is important to guarantee the security of RO PUF itself. In this paper, we propose a novel architecture of configurable RO PUF based on exclusive-or (XOR) gates. First, it dramatically increases the hardware efficiency compared with other types of RO PUFs. Second, it mitigates the vulnerability to EM analysis attack by placing the adjacent RO arrays in accordance with the cosine wave and sine wave so that the frequency of each RO cannot be detected. We implement our proposal in XINLINX A-7 field programmable gate arrays (FPGAs) and conduct a set of experiments to evaluate the quality of the responses. The results show that responses pass the National Institute of Standards and Technology (NIST) statistical test and have good uniqueness and reliability under different environments. Therefore, the proposed configurable RO PUF is suitable to establish a key extraction mechanism in a WSN.

## 1. Introduction

Wireless sensor networks (WSNs) are an emerging technology used in the internet of things (IoT) era where smart devices are connected and interact with each other [1]. Sensors act as tentacles of the network and are responsible for sensing the environment, collecting and communicating sensed data [2]. However, the unique characteristics of a WSN make security a challenging issue for the following reasons. First, the sensor nodes are generally physically exposed in an external environment with unstable temperature. Second, the digital secret keys in non-volatile memory can be extracted through semi-invasive and invasive attacks [3]. Third, conventional cryptographic solutions that depend on complicated crypto modules are impractical in a WSN because of the strict resource and power constraints.

In order to establish a highly efficient, leak-proof and tamper-proof key extraction mechanism in a WSN [4,5], physical unclonable function (PUF) [6] has been proposed in recent years. Due to the differences of manufacturing, PUF can be considered as an electronic fingerprint for which the same circuit on several devices will return different responses from an identical challenge. Since the challenge response pairs (CRPs) carry unique information on the underlying hardware variations, PUF is a potential option for key extraction in a WSN.

The PUFs targeting field programmable gate array (FPGA) can be categorized as memory-based and delay-based PUFs [7]. SRAM PUF [8] is a typical memory-based PUF that makes use of the uncertain transient characteristics of SRAM cells. Generally, a memory-based PUF cannot generate enough CRPs, so it is not suitable for key extraction, which needs sufficient random numbers. Arbiter PUF [9], ring oscillator (RO) PUF [10], and configurable ring oscillator (CRO) PUF [11] are representative delay-based PUFs. The output of an arbiter PUF is determined by comparing the propagation delay of two completely symmetrical transmission paths. However, an arbiter PUF suffers from poor uniqueness and is difficult to implement in FPGA [12]. RO PUF exploits the difference in frequency between two identical ROs [10] but requires significant hardware resources to generate enough CRPs for key extraction. In 2011, Maiti et al. [11] first proposed the CRO PUF to reduce the hardware requirements. Then, Zhang et al. [12] proposed an XOR gate-based CRO (XCRO) PUF that further improved the CRO PUF.

The major advantages of RO PUF are better uniqueness and reliability that make it capable of generating a bit stream with higher randomness in complicated environments. Due to the limited hardware resource of the sensor node and its physical exposure to attackers, the hardware efficiency and resistance to side-channel attacks should be considered when deploying RO PUF in a WSN. The motivation of this paper is two-fold. First, we need design RO PUF that achieves better performance with less hardware consumption to meet the strict constraints of the WSN. Second, the proposed RO PUF should resist side-channel attacks, especially localized electromagnetic (EM) attacks [13,14] that aim to clone the function of RO PUF though analyzing the frequency of each RO.

Contributions: In this paper, we design a novel architecture of CRO PUF based on XOR gates and multiplexers (MUXs), which consumes much less hardware resources to generate 1-bit response compared with other designs of CRO PUFs. In order to mitigate the vulnerability to EM attacks, we place adjacent ROs in accordance with the cosine wave and the sine wave. In this structure, the leaked electromagnetic fields of the two adjacent ROs will overlap so that the EM detector cannot distinguish the frequency of each RO. Finally, we implement the proposed CRO PUF on XILINX A-7 FPGA using hard macros and tool command language (TCL) to accurately place the look up tables (LUTs) and the MUXs. The experimental results show that our design has good randomness, uniqueness and reliability when the ambient temperature rises from 25 °C to 70 °C.

The rest of this paper is organized as follows: Section 2 briefly reviews the traditional RO PUFs and CRO PUFs. In Section 3, we present the architecture of our CRO PUF that features resistance to EM attack with less hardware consumption. In Section 4, we elaborate on how to implement the proposed CRO PUF in FPGA. The analysis of hardware efficiency, randomness, uniqueness, and reliability is provided in Section 5. Finally, Section 6 concludes the paper.

## 2. Related Work

### 2.1. Basic RO PUF

**Figure 1:**
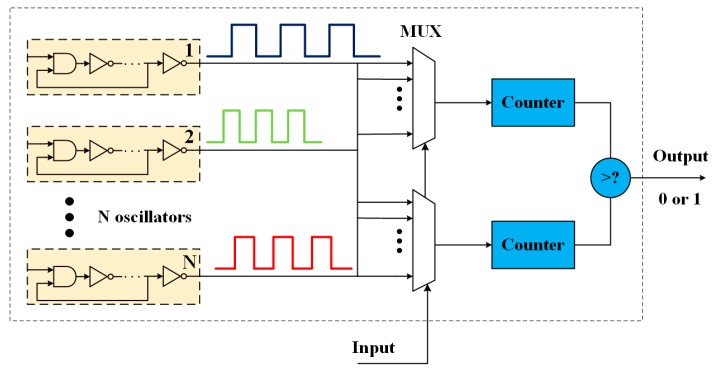
1-out-of-N ring oscillator-based physical unclonable function (RO PUF) architecture [10].

When an even number of inverters are connected in series and the enabling signal is set high, the RO will output a signal with specific frequency. The RO PUFs exploit the fact that the frequencies of identically laid-out ROs have random but static differences caused by the manufacturing process variations. The PUF’s output is generated by comparing the frequencies between the pair-wise ROs. Since the temperature has a significant impact on the frequency of RO, Suh et al. [10] proposed generation on a reliable unclonable bit by comparing the fastest and slowest ROs of N ROs. As shown in Figure 1, this 1-out-of-N RO PUF will be more reliable at the cost of huge hardware consumption.

Yin and Qu [15,16] proposed a group-based RO PUF with a longest increasing subsequence-based grouping algorithm (LISA) to enhance the hardware utilization. LISA is responsible for partitioning the ROs into groups, where every pair of ROs in each group will have a fixed relative frequency under different operating temperatures. Then, more bits can be generated according to the frequency orderings of the ROs in each group, which guarantees that the generated bits are reliable. The main drawback of group-based RO PUF is the requirement to measure each RO’s speeds at the lowest and highest operating temperatures.

### 2.2. Configurable RO PUF

**Figure 2:**
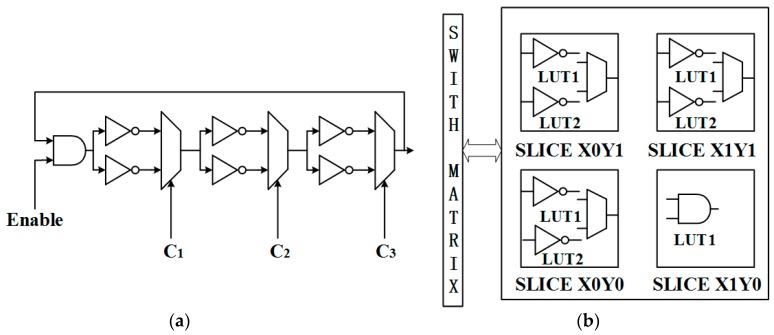
(**a**) Configurable RO (**b**) The configurable RO fits in a single configurable logic block (CLB) on a Xilinx Spartan 3E platform [11].

Maiti et al. [11] first proposed a RO PUF based on configurable ROs in 2011. Every RO is restricted to one configurable logic block (CLB) of XILINX FPGA, as shown in Figure 2. This size constraint has a major advantage in that the resources within the CLB are routed restrictedly to the switch box associated with that CLB. This RO can be defined as a hard macro so that all routing and logic resources will remain identical when duplicated. There are four slices in one CLB of XILINX Spartan 3 FPGA. In one slice, there are two LUTs, one MUX, two flip-flops etc. C_1_, C_2_ and C_3_ are configuration signals that choose which LUT will be used as the inverter. Thus, it can produce eight distinct ROs with eight distinct configurations; each RO has different frequency property under a specific configuration. When comparing a pair of ROs, their configuration should be the same so that the difference of frequencies is determined by manufacturing process variations instead of routing variations. In Maiti’s design, two ROs take up one CLB and can generate eight bits.

**Figure 3:**
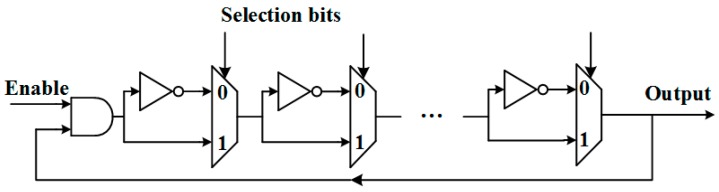
Architecture of the configurable RO [17].

In 2014, Gao et al. [17] proposed a novel construction of a configurable RO. Figure 3 illustrates the architecture with the flexibility to select inverters for constructing the ROs. In each stage, there is a multiplexer for determining whether the inverter will be used in the RO, which is implemented by the selection bit of the multiplexer. For example, if the selection bit is “1”, the corresponding inverter will be included in the RO. Otherwise, the inverter will not be included so that the signal will go through the wire to the next stage with negligible delay. The term configuration vector is used to refer to the collection of all the multiplexer selection bits. The experimental results demonstrate that the framework improves the security and reliability of RO PUF secrecy. Moreover, it achieves four times more hardware efficiency than the robust 1-out-of-8 RO PUF.

**Figure 4:**
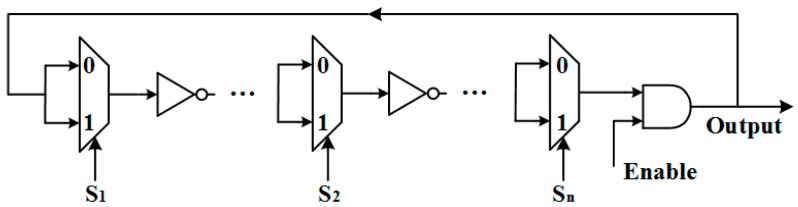
Architecture of the reconfigurable RO [18].

In 2016, Cui et al. [18] proposed a reconfigurable RO (RRO) PUF by further simplifying the hardware complexity. As shown in Figure 4, only an inverter and a multiplexer are used in each delay unit. The selection nodes {S_1_, S_2_, … S_n_} of the multiplexers act as configurable nodes to determine the transmission path to form a RO. This structure has an enhanced capability to generate a large number of bit responses with very good uniqueness and reliability.

In summary, CRO PUFs are becoming the most promising research field in PUFs because they have good PUF properties in terms of uniqueness and reliability. However, most research focuses on improving hardware efficiency but ignores the resistance to side-channel attacks, which is one of the major concerns in the deployment of CRO PUF in a WSN.

## 3. Architecture of Anti-EM Attack CRO PUF

**Figure 5:**
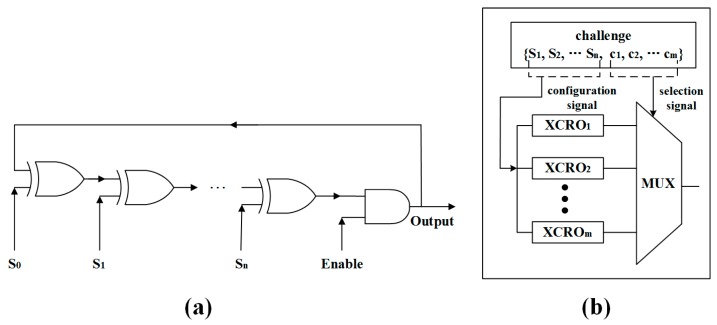
(**a**) Architecture of an XOR-gate-based configurable RO (**b**) Challenge signals of an XOR-gate-based configurable RO PUF [12].

Zhang et al. [12] presented an XOR gate-based configurable RO (XCRO) in 2017. As shown in Figure 5a, the XOR gate acts as a wire when S_m_ = 0, and the XOR gate acts as an inverter when S_m_ = 1. For instance, in a four selection scheme {S_1_, S_2_, S_3_, S_4_}, there will be eight different ROs: {0,1,1,1}, {1,0,1,1}, {1,1,0,1}, {1,1,1,0}, {1,0,0,0}, {0,1,0,0}, {0,0,1,0}, and {0,0,0,1}. More ROs means more responses under the same configuration signal. As shown in Figure 5b, the n-bit challenge {S_1_, S_2_, ... S_n_} will be used as the configuration signal and the m-bit challenge {c_1_, c_2_, … c_m_} will be used to select XCROs to generate a response.

**Figure 6:**
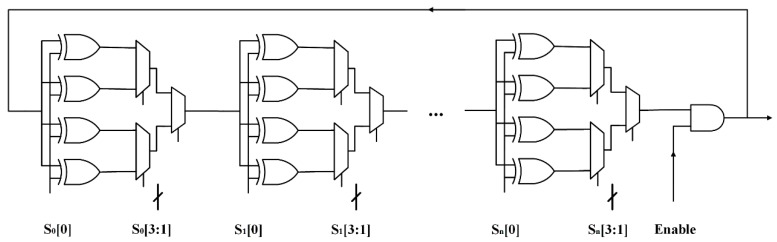
Architecture of the proposed configurable RO.

Inspired by the XCRO, we have designed a novel CRO architecture based on XOR gates and MUXs that is able to generate more CRPs with the same hardware resources, which is illustrated in Figure 6. Each delay unit of our proposed CRO PUF consists of four XOR gates and three MUXs. S_m_ [3:1] is responsible for choosing which XOR gate will be used in the m_th_ delay unit and S_m_ [0] determines the chosen XOR gate to act as a wire or an inverter. In this way, the RO array contains a large number of different ROs with different configuration signals. For example, there will be C_7_^5^ × 4^5^ = 21,504 choices if we need a RO with five inverters in a RO array that contains seven delay units. First, the number of choices is C_7_^5^ when we choose five delay units from seven to act as inverters. Second, there are four XOR gates for each delay unit, so we have 4^5^ choices to decide the five specific inverters to form the RO.

Compared with XCRO, the major advantage of our proposal is the higher hardware efficiency, which will be evaluated in Section 5. Moreover, our proposed CRO array is more flexible because there can be more inverters in a RO than XCRO that has only seven inverters at most.

The resistance to EM attack was not taken into account in XCRO PUF. Merli et al. presented analysis methods to measure the EM emission frequency and map it to the corresponding ROs [19]. They practically proved that it is possible to recover the response bits generated by RO PUF with this approach. In [13], Merli et al. continued to demonstrate that it is feasible to locate and measure the EM emission of a single tiny RO implemented within a single CLB of a Xilinx Spartan-3A that consists of only three inverters. They also presented a localized EM attack targeting standard and protected RO PUFs.

There were two major methods to protect RO PUFs against side-channel analysis. First, every RO is used only once in a single comparison to prevent RO identification. This structure doubled hardware overhead because it could only extract n/2 response bits from n ROs. Second, they compared all n ROs at the same time to resist the side-channel attack and m-1 response bits could still be extracted. However, every RO needs its own counter, which brought about unacceptable hardware overhead.

**Figure 7:**
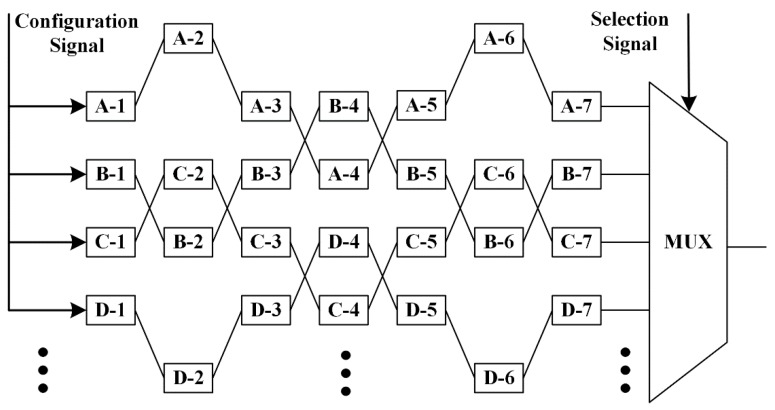
Architecture of the anti-electromagnetic (EM) attack CRO PUF.

In this paper, we propose a novel architecture of the anti-EM attack CRO PUF. According to [13], a successful attack requires the separated areas of leakage to be located. The attacker should pinpoint the locations which show a significant standard deviation in the frequency domain. For the purpose of mitigating the vulnerability to EM attack, we make it much more difficult for the attacker to distinguish the frequency of each RO. As shown in Figure 7, we place one RO in accordance with the cosine wave and place the adjacent RO in accordance with the sine wave, which can be easily implemented in FPGA. In this way, the EM emission of adjacent ROs will overlap and act as noise so that the attacker cannot extract the frequency of the target RO even if its exact location is known. The anti-EM attack CRO PUF will not bring about routing difference for ROs because routing modes of adjacent ROs are a mirror image.

## 4. FPGA Implementation

**Figure 8:**
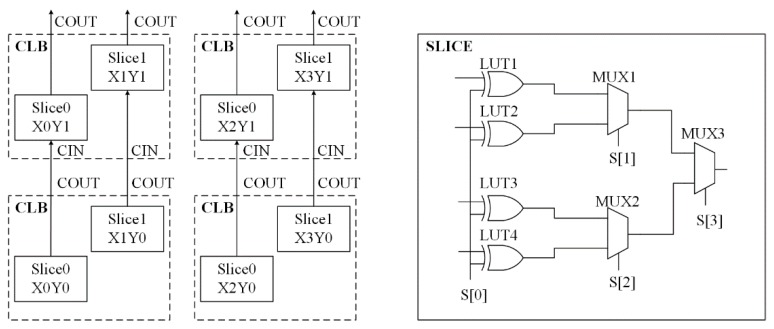
Hardware resource of Xilinx A-7 field programmable gate arrays (FPGA).

The proposed anti-EM attack CRO PUF is implemented in Xilinx A-7 FPGA. The hardware resource is illustrated in Figure 8. Each CLB has two slices and each slice has 4 LUTs, 3 MUXs, and 8 D-flip-flops [20]. The LUTs can be used as different types of combinational circuits, including an AND gate and XOR gate. Thus, four XOR gates of the delay unit are implemented by the four LUTs and three MUXs determine which XOR gate will be used. In our design, four CLBs are required to implement a CRO array with seven delay units, which can generate N ROs.

N = 4^7^ × C_7_^7^ + 4^5^ × C_7_^5^ + 4^3^ × C_7_^3^ + 4 × C_7_^1^ = 40,156 (1)

**Figure 9:**
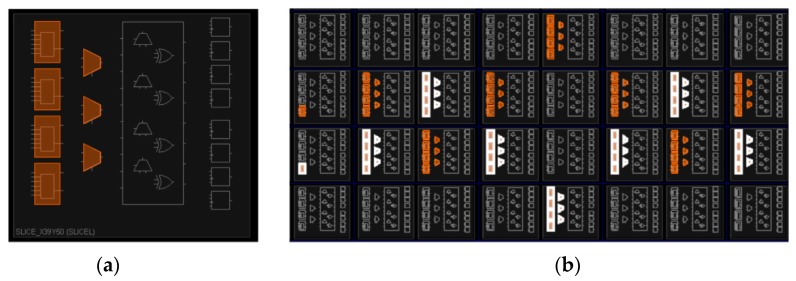
(**a**) Elements in a single slice; (**b**) Two adjacent ROs.

As shown in Figure 9a, hard macros and tool command language (TCL) are used to accurately place the XOR gates and the MUXs of each delay unit into one slice, which makes full use of the hardware resource of FPGA. All the routing in the slice is physically connected, so the routing difference of four XOR gates can be ignored. In Figure 9b, two adjacent CROs are placed in accordance with the cosine wave and the sine wave to enhance the resistance against EM attack.

## 5. Experimental Results

Considering that the sensor nodes are physically exposed in a different operating environment and have limited hardware resources, four criteria of CRO PUF should be taken into account for the purpose of establishing a key extraction mechanism in a WSN: hardware efficiency, randomness, uniqueness, and reliability. We will first briefly explain how to quantify these criteria and then conduct a series of experiments to demonstrate that the proposed CRO PUF meets all the conditions for deployment in a WSN.

**Hardware efficiency**. Since CLB is the basic component of XILINX FPGAs, the hardware efficiency of PUFs can be quantified by the number of required CLBs when generating the same number of responses. In other words, if a PUF design uses the same number of CLBs to generate more responses, it is more hardware efficient.

**Randomness**. The first step of a key extraction mechanism is to condition the outputs of CRO PUF. (The conditioning operation consists of hashing, compressing, etc., which is out of the scope of this paper.) To guarantee the security of the extracted keys, the outputs of CRO PUF should be truly random. The entropy of PUF-response bit streams is the major metric to quantify the randomness.

**Uniqueness**. Uniqueness is the difference between the outputs of PUFs implemented in different chips under the same challenge. Good uniqueness ensures that the keys extracted from different chips are independent. The intra Hamming distance is used as the metric to quantify uniqueness.

**Reliability**. The PUF is reliable if its response remains the same under the same challenge even though the operating environment changes, which ensures the stability of the key extraction mechanism. The reliability is important for sensor nodes because they are generally working in an environment with unstable temperature. The inter Hamming distance is used as the metric to quantify reliability.

### 5.1. Hardware Efficiency

In this paper, we use the same metric as introduced in [12] to measure the hardware efficiency of different RO PUFs, i.e., the average number of CLBs generated per response bit:

E = N/B_response_ (2)

E denotes the hardware efficiency of RO PUF, B_response_ denotes the number of response bits, and N denotes the number of required CLBs to generate B_response_ bits. Thus, the higher the hardware efficiency of RO PUF is, the less the value of E will be. We will compare the hardware efficiency E among basic RO PUF [10], CRO PUF [17], RRO PUF [18], XCRO PUF [12], and our proposed CRO PUF.

In basic RO PUF [10], each RO has a fixed odd number of inverters that can be implemented by one CLB. At most, n CLBs can generate Cn^2^ response bits by comparing all pairs of ROs. Therefore, the hardware efficiency of basic RO PUF is:

E_RO_ = n/Cn^2^ = 2n/n (n − 1) (3)

In CRO PUF [17] and RRO PUF [18], one CLB can generate eight different ROs. Theoretically, a pair of ROs with the same configuration but in different CLBs can output one response bit. Therefore, the hardware efficiency of CRO PUF and RRO PUF is:

E_CRO_ = E_RRO_ = n/8 × Cn^2^ = 2n/8 × n (n − 1) (4)

In XCRO PUF [12], one CLB can generate 64 different ROs. n CLBs can generate 64 × Cn^2^ distinct pairs of ROs. Therefore, the hardware efficiency of XCRO PUF is:

E_XCRO_ = n/64 × Cn^2^ = 2n/64 × n (n − 1) (5)

In our proposed CRO PUF, instead of implementing eight different ROs in one CLB—as is the case for XCRO PUF—we use four CLBs to generate a RO array. As elaborated in Section 3, there are seven stages for a RO array and there are four XOR gates in each stage. We can set the configuration signals to choose 1 out of 4 XOR gates and determine it as an inverter or a wire. For ROs with seven inverters, there will be 4^7^ choices. Thus, for a RO array, there will be 4^7^ × C_7_^7^ + 4^5^ × C_7_^5^ + 4^3^ × C_7_^3^ + 4^1^ × C_7_^1^ = 40,156 different ROs. Then, 4 × n CLBs can generate 40,156 × Cn^2^ distinct pairs of ROs. Therefore, the hardware efficiency of XCRO PUF is:

E = 4 × n/40,156 × Cn^2^ = 2n/10,039 × n (n − 1) (6)

**Figure 10:**
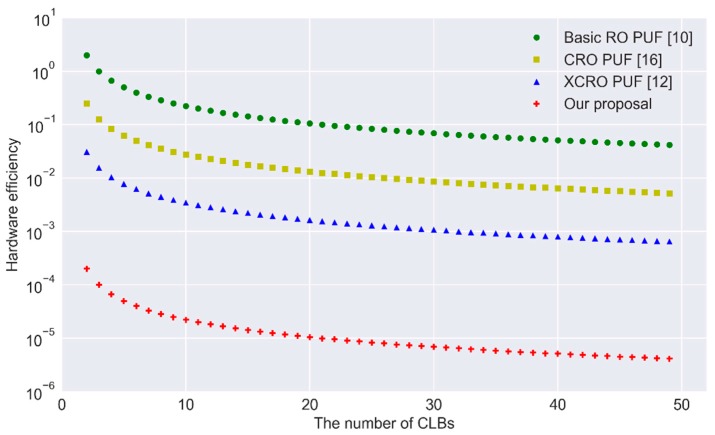
Hardware efficiency of different types of RO PUFs.

As shown in Figure 10, our proposal significantly increases the hardware efficiency.

### 5.2. Randomness

The unpredictability of RO PUF’s responses is measured by randomness that determines whether the RO PUF is biased or not [21]. The key extraction mechanism in a WSN needs different responses under different challenges without measurable trend. Ideally, half of the response bits of an unbiased RO PUF should be altered if one bit in a challenge is changed. The distiller technique [22] will be applied in our design to filter out the systematic variation. In this paper, we measure the randomness of the proposed CRO PUF using NIST’s statistical test suite [23], which is generally employed to evaluate the quality of randomness for RO PUF’s responses used for cryptographic applications. For comparison, we will use the similar approach found in [17] to evaluate the randomness of bit streams generated by basic RO PUF [10], CRO PUF [17], and our proposal. In the experiment, each RO is configured with five inverters which is the same as in [17]. Limited by the minimum length of response in some tests, we perform 8 out of 15 tests. We implement 15 RO arrays that can generate streams containing C_15_^2^ = 105 bits and combine 10 independent responses as an input to meet the length requirement of the Discrete Fourier Transform test. The pass rate of three different RO PUFs for each statistical test is listed in Table 1. Although CRO PUF [17] shows better results in terms of randomness, our proposal also passes the NIST test with higher hardware efficiency. The results imply that the proposed CRO PUF has good randomness that meets the requirement of the key extraction mechanism in a WSN.

### 5.3. Uniqueness

The uniqueness of the RO PUF response is one important factor that determines the quality of the RO PUF. It is obvious that the PUF is unusable if different PUFs generate identical or very similar responses with the same challenge. If the uniqueness of PUF responses is good enough and logic-0 and logic-1 distribute uniformly in response, the expectation of Hamming distance (HD) between the PUF responses should be 50%. Generally, HD is used to measure the uniqueness of the PUF response. For a pair of n-bit PUF responses: P*_i_* and P*_j_* (i≠j), the HD is the number of bits that P*_i_* is different from P*_j_*. Then, the inter-chip variation is defined as an average percentage of HD between any pairs out of k PUF outputs. Equation 1 [24] is as follows.

(7) $$\mathrm{Inter\_var}=\frac{\sum_{i=0}^{k-1}\sum_{j=0,i\neq j}^{k-1}\frac{HDa_{ij}}{c\times (r-1)}\times 100\%}{k\times (k-1)}$$

In the formula, HDa_i,j_ represents the HD of PUF outputs between a pair of n-bit PUF responses: P*_i_* and P*_j_*, where:

(8) $$HDP_{ij}=\sum_{m=1}^{n}\left(r_{i,m}⊗r_{j,m}\right)$$

Under ideal conditions, the average value of inter-chip variation equals 50%, which means that there is no correlation between the outputs of different PUFs in different chips. In other words, the uniqueness is better if the inter-chip variation is closer to 50%. In our experiment, we implement four proposed CRO PUFs in different areas in one FPGA so that we get 20 CRO PUFs out of five FPGAs. Then, we calculate the inter-chip variation of the 20 responses. Obviously, if the average inter-chip variation of PUFs in different locations of the same chip is close to 50%, the value will be closer to 50% when different RO PUF is implemented in a different chip.

Since we have 20 different CRO PUFs, there are 20 different responses for each challenge. We randomly choose 256 different challenges and each challenge will produce C_20_^2^ = 190 HDs out of 20 responses. In order to increase the efficiency of the experiment, we send challenges and received responses through a serial port and use MATLAB to calculate the result. In this way, the experiment was carried out automatically for one CRO PUF. We just need to change CRO PUF and execute MATLAB functions.

**Figure 11:**
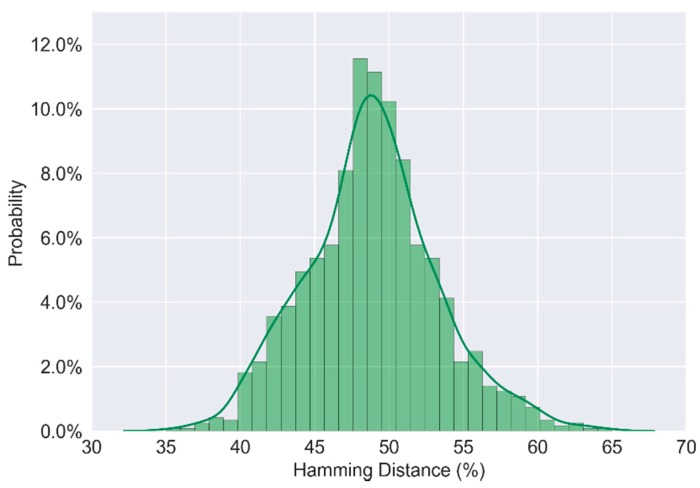
Uniqueness of responses.

The experimental result is shown in Figure 11. From this frequency histogram, the average value of inter-chip variation is 48.85%.

### 5.4. Reliability

Reliability is used to evaluate the stability of PUF responses in different environments. Ideally, responses of RO PUF should remain identical with the same challenge. Intra-chip variation is defined to measure the reliability of RO PUFs. We generate one output from the same RO PUF with the same configuration; the value of intra-chip variation is the average Hamming distance between any pair of regenerated outputs. Equation (2) [24] is as follows:

(9) $$\mathrm{Inter\_var}=\frac{\sum_{p=0}^{l-1}\sum_{q=0,p\neq q}^{l-1}\frac{HDb_{pq}}{c\times (r-1)}\times 100\%}{l\times (l-1)}$$

In the formula, HD, b, p, q represents the Hamming distance between the pth and qth regenerated output of the same RO PUF out of one regenerated PUF output. Under ideal conditions, the average value of intra-chip variation equals 0%, which means that there is no difference between the responses of the same PUF under the same challenge. In other words, the reliability is better if the intra-chip variation is closer to 0%. However, PUF responses may be unstable because external factors will affect the circuit delay and change the frequency of the RO. Among several factors of influence appearing in the practical scenario, the ambient temperature variation is relatively more important because it can affect the circuit delay directly. Thus, we choose the temperature variation as the environmental factor to evaluate the performance of the proposed CRO PUF in this paper.

**Figure 12:**
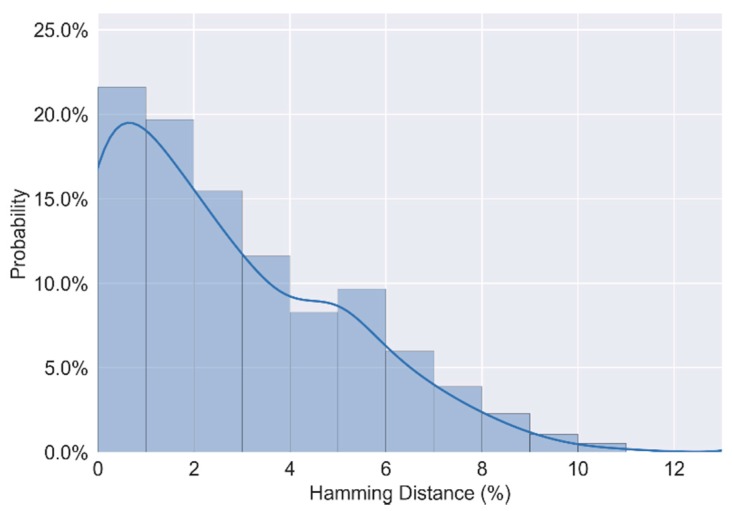
Reliability of responses.

Two sets of experiments were conducted. First, the proposed CRO PUF ran at about 25 °C We input 256 different challenges to CRO PUF and obtained 256 105-bit responses. Then we put FPGA in the thermotank with a temperature of 70 °C and conducted the same experiment. We calculated the Hamming distance between two responses with the same challenge under a different temperature. As shown in Figure 12, when the ambient temperature changes from 25 °C to 70 °C, the average value of intra-chip variation is 2.63%, the maximum is 11% and the minimum is 0%.

The average uniqueness and reliability of several representative PUFs are presented in Table 2. The results shows that the uniqueness of our proposal is better than basic RO PUF and conventional CRO PUF and the reliability is better than SRAM PUF. Therefore, the quality of our proposed CRO PUF is good enough to be exploited in a WSN.

## 6. Conclusions

In order to guarantee the security of a WSN, we have proposed a novel architecture of CRO PUF based on XOR gates and MUXs. Considering the limited resources of sensor nodes and the physical exposure to attackers, our design focuses on improving the hardware efficiency and enhancing the resistance to EM analysis attacks. We used four CLBs to implement a CRO array with seven delay units. Each delay unit consists of four XOR gates and three MUXs that make full use of the hardware resources of FPGA. We can arbitrarily choose one XOR gate out of four and determine whether it acts as an inverter or wire. Therefore, the number of distinct ROs is dramatically increased so that there will be many more CRPs with the same hardware consumption. To prevent the EM detector from measuring the frequency of each RO, we placed the CRO arrays in accordance with the cosine wave and sine wave. As a result, the frequency of the adjacent ROs will overlap to thwart EM analysis attack.

The responses’ randomness, uniqueness, and reliability are the major considerations when deploying PUF in a key generation mechanism for a WSN. A set of experiments was conducted to evaluate the quality of our proposal. First, the bit streams generated by the proposed CRO PUF passed the NIST statistical test, which is used to test the randomness of PUF’s responses for cryptographic applications. Second, the inter-chip variation (average is 48.85% and minimum is 35%) shows that our design has good uniqueness. Third, the intra-chip variation under different environments (average is 2.63% and maximum is 11%) shows that our design has good reliability. Therefore, our proposal meets the requirements of hardware efficiency, randomness, uniqueness, and reliability and is practical for use in a key generation mechanism for a WSN.

## Figures and Tables

**Table 1:** Success ratio of three types of RO PUFs in the NIST randomness test.

NIST Algorithms	Success Ratio (%)		
	Basic RO PUF [10]	CRO PUF [17]	Our Proposal
Frequency	96	99	98
Block Frequency	96	100	99
Runs	97	100	100
Longest Runs	97	100	100
Discrete Fourier Transform	94	97	96
Serial	96	99	96
Approximate Entropy	97	98	97
Cumulative Sums	98	99	98

**Table 2:** Average uniqueness and reliability of different types of PUF implemented in FPGA.

PUF Designs	Uniqueness (%)	Reliability (%)
SRAM PUF [8]	49.97	3.57
Basic RO PUF [10]	46.15	0.48
CRO PUF [11]	47.31	0.86
RRO PUF [18]	49.97	2.60
XCRO PUF [12]	48.76	2.28
Proposed CRO PUF	48.85	2.63
